# Electrochemical Sensor Based on ZnFe_2_O_4_/RGO Nanocomposite for Ultrasensitive Detection of Hydrazine in Real Samples

**DOI:** 10.3390/nano12030491

**Published:** 2022-01-29

**Authors:** Somayeh Tajik, Mohammad Bagher Askari, Sayed Ali Ahmadi, Fraiba Garkani Nejad, Zahra Dourandish, Razieh Razavi, Hadi Beitollahi, Antonio Di Bartolomeo

**Affiliations:** 1 Research Center of Tropical and Infectious Diseases, Kerman University of Medical Sciences, Kerman P.O. Box 76169-13555, Iran; s.tajik@kmu.ac.ir; 2 Environment Department, Institute of Science and High Technology and Environmental Sciences, Graduate University of Advanced Technology, Kerman P.O. Box 76318-85356, Iran; mbaskari@phd.guilan.ac.ir; 3 Department of Chemistry, Kerman Branch, Islamic Azad University, Kerman P.O. Box 763151-31167, Iran; saahmadi@iauk.ac.ir; 4 Department of Chemistry, Faculty of Science, Shahid Bahonar University of Kerman, Kerman P.O. Box 76169-13439, Iran; f.garkani95@gmail.com (F.G.N.); z.dourandish2017@gmail.com (Z.D.); 5 Department of Chemistry, Faculty of Science, University of Jiroft, Jiroft P.O. Box 78671-55311, Iran; R.Razavi@ujiroft.ac.ir; 6 Department of Physics “E.R. Caianaiello”, University of Salerno, 84084 Fisciano, Italy

**Keywords:** hydrazine, screen printed electrode, ZnFe_2_O_4_/RGO nanocomposite, voltammetry

## Abstract

We have developed a highly sensitive sensor of ZnFe_2_O_4_/reduced graphene oxide (ZnFe_2_O_4_/RGO) nanocomposite for electrochemical detection of hydrazine, fabricated by a simple hydrothermal protocol. Subsequently, a screen-printed electrode (SPE) surface was modified with the proposed nanocomposite (ZnFe_2_O_4_/RGO/SPE), and revealed an admirable electrocatalytic capacity for hydrazine oxidation. The ZnFe_2_O_4_/RGO/SPE sensor could selectively determine micromolar hydrazine concentrations. The as-produced sensor demonstrated excellent ability to detect hydrazine due to the synergistic impacts of the unique electrocatalytic capacity of ZnFe_2_O_4_ plus the potent physicochemical features of RGO such as manifold catalytic sites, great area-normalized edge-plane structures, high conductivity, and large surface area. The hydrazine detection using differential pulse voltammetry exhibited a broad linear dynamic range (0.03–610.0 µM) with a low limit of detection (0.01 µM).

## 1. Introduction

Hydrazine (N_2_H_4_) is an agent with broad-spectrum applications such as reducing agents, emulsifiers, catalysts, antioxidants, and corrosion inhibitors, and as precursors to produce various explosives, dyestuffs, pesticides, herbicides, insecticides, and pharmaceutical derivatives. On the other hand, the excessive use of this agent can generate toxicity and irreversible cell damage, and develop complications such as brain and liver dysfunction, DNA damage, blood abnormalities, and irreversible neuronal deterioration. According to the Occupational Safety and Health Administration (OSHA) and the National Institute for Occupational Safety and Health (NIOSH), the hydrazine density in the air of the workplace should not exceed 0.03 mg/mL for 1 h exposure [1,2,3]. Accordingly, the hydrazine content needs to be accurately determined in clinical samples and in a cost-effective manner.

The hydrazine content has been previously detected by different techniques, including spectrophotometry [4], chemiluminescence [5] and gas chromatography [6]. Such techniques involve complex processes with relatively narrow linear ranges and low accuracy. Electrochemical methods are fast, highly sensitive, selective, cost-effective and enable portable devices [7,8,9,10,11,12,13,14]. Hence, we select these methods for hydrazine detection in the present study.

Nevertheless, the kinetics indicate that the electrochemical hydrazine oxidation is slow, and unmodified electrodes need fairly high overpotentials [15,16,17]. Various techniques have been applied in the attempt to minimize the problem of high overpotentials. Over the past two decades, many efforts have been made to further control the chemical behavior of electrodes through chemical modified electrodes (CMEs). An ideal CME should be reportedly produced according to three main factors, including admirable current response to target molecules, simple and cost-effective fabrication process, and high selectivity, sensitivity, and stability [18,19,20,21,22,23,24,25].

Screen printing is a method widely used for microelectronics to construct various electrochemically disposable sensing electrodes. Screen-printed electrodes (SPEs) are versatile, cost-effective, and simple analytical tools, suitable for miniaturization and useful for chemical electroanalysis [26,27,28,29,30]. Electrode surface modification has been recently performed for detection of target molecules using various nanomaterials. The modification of the SPE surface has been carried out exploiting different nanomaterials to promote the electrochemical behaviors [31,32,33,34,35].

Further attention has been given to the spinel ferrites (with a general molecular formula of MFe_2_O_4_, M=Co, Ni, Zn and Cd) owing to their strong physical, catalytic, chemical and magnetic activities. The synergistic impact of Fe and Zn in zinc ferrite (ZnFe_2_O_4_) enhances the redox process in electrochemistry, making ZnFe_2_O_4_ suitable for use in different devices such as solar cells, batteries and electrochemical sensors. ZnFe_2_O_4_ has high availability, low price, lower toxicity, green application, potent electrochemical response, and large surface area [36,37,38,39,40]. Moreover, incorporation of ZnFe_2_O_4_ in conductive carbon-based materials, such as reduced graphene oxide, is a way to reach an excellent electrochemical response. This nanostructure has also been used in various fields such as sensing, due to its unique properties such as large surface area, electron mobility at ambient temperature, admirable electrical conductivity, flexibility, and strong mechanical features [41,42,43,44,45].

The present work aimed to fabricate ZnFe_2_O_4_/RGO nanocomposite through a facile hydrothermal protocol to detect hydrazine ultra-sensitively in the water specimens. The synergic impact of metal nanoparticles plus graphene was expected to be effective in ZnFe_2_O_4_/RGO nanocomposite. The SPEs were selected to fabricate working electrodes owing to cost-effectiveness, reproducibility, simple production process, and flexibility. The proposed ZnFe_2_O_4_/RGO/SPE sensor was examined as well for its applicability to detect the hydrazine in the water specimens.

## 2. Materials and Methods

### 2.1. Chemicals and Equipment

The electrochemical measurements were performed by a PGSTAT 302N Autolab potentiostat/galvanostat analyzer (Eco-Chemie, Utrecht, The Netherlands). All test conditions were monitored by General Purpose Electrochemical System (GPES) software. A three-part DropSens SPE (DRP-110, Metrohm DropSens, Oviedo, Spain) included a graphite working electrode, a silver pseudo-reference electrode, and a graphite auxiliary electrode. The solution pH values were measured by a Metrohm 710 pH meter. X-ray diffraction analyses were performed with a Thermo Scientific device (ARL EQUINOX 3000, (Thermo, Waltham, MA, USA)). FE-SEM images were obtained using a Hitachi Model S-3700N (Hitachi, Tokyo, Japan). The TEM images were obtained using a Phillips EM 2085 machine (Philips, Amsterdam, The Netherlands).

All reagents possessed analytical grade and were from Merck (Darmstadt, Germany). Orthophosphoric acid and related salts were utilized to prepare all buffer solutions at the pH values (2.0 to 9.0).

### 2.2. Fabrication of ZnFe_2_O_4_/RGO Nanocomposite

The GO synthesis was performed by the modified Hummers’ method. Initially, ZnFe_2_(C_2_O_4_)_3_ was produced to fabricate ZnFe_2_O_4_ nanorods via a hydrothermal method. Thus, FeSO_4_·7H_2_O (0.556 g) and ZnSO_4_·7H_2_O (0.288 g) were dissolved in deionized water (15 mL) while stirring on a magnetic stirrer, followed by adding ethylene glycol (45 mL) containing H_2_C_2_O_4_ (3 mM). The obtained mixture was stirred for 10 min to give a yellow solution. Then, the resulting solution was transferred into a reactor (80 mL), heated to 120 °C for 24 h, cooled down to lab temperature, and centrifuged to collect yellow precipitate of ZnFe_2_(C_2_O_4_)_3_. The product was rinsed thoroughly with deionized water and ethanol and placed in a vacuum oven at 60 °C for 2 h for dehydration. The treatment of the achieved powder was performed by heating at 350 °C for 30 min to collect ZnFe_2_O_4_ nanorods with the ramping rate of 10 °C/min [46]. For the synthesis of ZnFe_2_O_4_-RGO, as mentioned in the synthesis of ZnFe_2_O_4_, 3 mg of GO was added to the precursors of the synthesis of ZnFe_2_(C_2_O_4_)_3_, namely FeSO_4_·7H_2_O (0.556 g) and ZnSO_4_·7H_2_O (0.288 g), and the precursors were then dissolved in 15 mL of deionized water with a magnetic stirrer. The other steps were the same as the synthesis of ZnFe_2_O_4_. It should be noted that in this step and in hydrothermal operation, GO is also converted to RGO.

### 2.3. Fabrication of Modified Electrode

A facile protocol was performed to cover a bare SPE by the ZnFe_2_O_4_/RGO nanocomposite. Specifically, 1 mg of ZnFe_2_O_4_/RGO nanocomposite was dispersed in 1 mL aqueous solution and ultra-sonicated for half an hour; then, 4 µL of the produced suspension was poured dropwise on the surface of SPE working electrode. Finally, the obtained solution was air-dried.

### 2.4. Real Sample Analysis

The real specimens included river, drinking, and tap water samples, which were filtered thoroughly before analysis and diluted with 0.1 M PBS with dilution factor of 1:4. Then different hydrazine concentrations were added to the specimens and analysis with standard addition method [9].

## 3. Results

### 3.1. Characterisation of the ZnFe_2_O_4_/RGO Nanocomposite

The XRD spectra were taken for the determination of the purity and crystallinity of as-produced nanomaterials, the results of which are shown in Figure S1a. According to this diffraction pattern, the peaks marked at angles of about 74°, 62°, 57°, 53.5°, 42°, 35° can be related to ZnFe_2_O_4_, which is in full compliance with JCPDS (10-22-22). A wide peak is also seen around the 25° angle, which belongs to RGO.

The TEM and SEM images were applied to explore the surface morphology and thicknesses of fabricated samples. The SEM images of ZnFe_2_O_4_-RGO (Figure S1b) show that ZnFe_2_O_4_ nanorods are uniformly placed on the surface of the reduced graphene oxide nanosheets. The TEM image of the ZnFe_2_O_4_-RGO (Figure S1c) also shows ZnFe_2_O_4_ nanorods dispersed on the surface of a highly transparent RGO nanosheet.

### 3.2. Electrochemical Responses of Hydrazine on the Surface of ZnFe_2_O_4_/RGO/SPE

**Figure 1:**
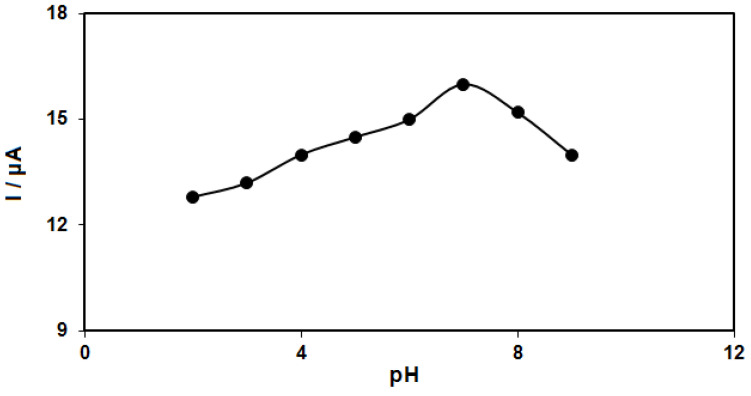
Plot of Ip vs. pH obtained from DPVs of ZnFe_2_O_4_/RGO/SPE in a solution containing 250.0 μM of hydrazine in 0.1 M PBS with different pHs (2.0, 3.0, 4.0, 5.0, 6.0, 7.0, 8.0, and 9.0).

The solution pH values influence the electrochemical responses of hydrazine (Equation (1)), highlighting the necessity for optimizing the solution pH to determine the electrocatalytic hydrazine oxidation, which was evaluated in 0.1 M PBS at various pH values (2.0 to 9.0) on the ZnFe_2_O_4_/RGO/SPE surface using differential pulse voltammetry. The results suggested a neutral pH value to achieve the best outcomes of hydrazine electrooxidation on the ZnFe_2_O_4_/RGO/SPE surface (Figure 1). Hence, the optimal pH value was selected to be 7.0 for this purpose in the next tests.

N_2_H_4_ + 2H_2_O → 2NH_2_OH + 2H^+^ + 2e^−^ (1)

**Figure 2:**
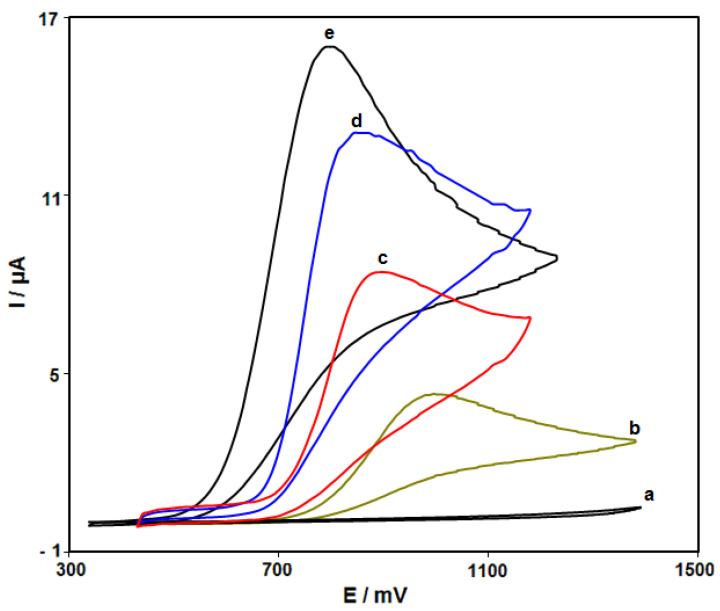
Cyclic voltammograms (CVs) recorded for electrooxidation of hydrazine. Curve a is the CV of bare SPE in 0.1 M PBS at the pH value of 7.0. Curves b–e are CVs at the surfaces of bare SPE, ZnFe_2_O_4_/SPE, RGO/SPE and ZnFe_2_O_4_/RGO/SPE in the presence of 0.1 M PBS at the pH value of 7.0 for the detection of hydrazine (250.0 μM). In all cases, the scan rate is 50 mV/s.

Figure 2 shows the cyclic voltammograms (CVs) recorded for electrooxidation of hydrazine (250.0 μM) on the surfaces of bare SPE, ZnFe_2_O_4_/SPE, RGO/SPE, and ZnFe_2_O_4_/RGO/SPE. The findings from the CVs confirmed the best hydrazine oxidation on the ZnFe_2_O_4_/RGO/SPE surface at 800 mV, about 200 mV more negative than that on the bare SPE, underlining a significant improvement in hydrazine oxidation signal via the ZnFe_2_O_4_/RGO nanocomposite. Moreover, ZnFe_2_O_4_/RGO/SPE in buffer solution showed no anodic or cathodic peak.

### 3.3. Results of Scan Rate Impact

**Figure 3:**
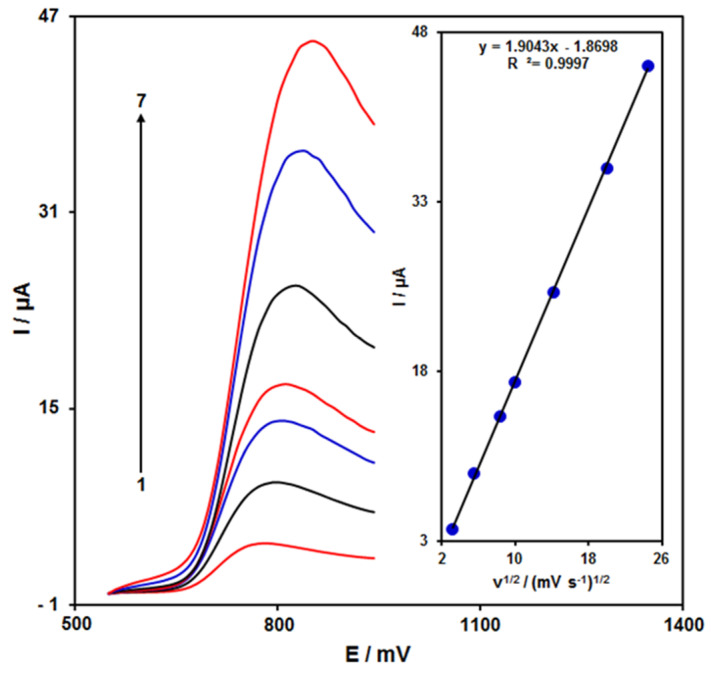
LSVs of ZnFe_2_O_4_/RGO/SPE in the presence of 0.1 M PBS at the pH value of 7.0 for detection of hydrazine (150.0 μM) at different scan rates, indicated by numbers 1–7 corresponding to 10, 30, 70, 100, 200, 400, and 600 mV/s. Inset: anodic peak current variation versus ν^1/2^.

Figure 3 shows the scan rate impact on the hydrazine oxidation current, the results of which indicated an increase in the peak current with increasing scan rate. The oxidation process followed the diffusion-limited reactions obtained from the linear dependence of the anodic peak current (Ip) on the square root of the scan rate (ν^1/2^, 10–600 mV/s).

### 3.4. Chronoamperometric Measurements

**Figure 4:**
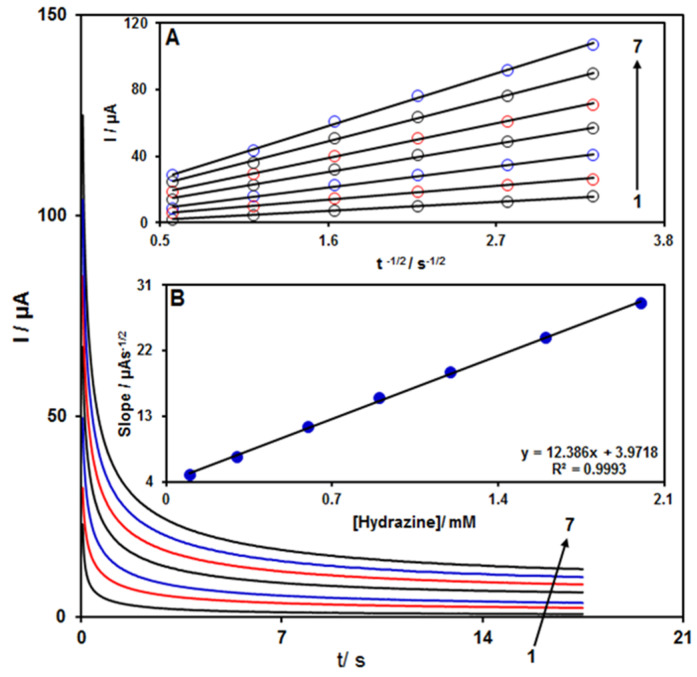
Chronoamperograms for ZnFe_2_O_4_/ RGO/SPE in the presence of 0.1 M PBS at the pH value of 7.0 for detection of hydrazine at different concentrations, indicated by numbers 1–7 corresponding to 0.1, 0.3, 0.6, 0.9, 1.2, 1.6, and 2.0 mM of hydrazine. Insets: (**A**) Cottrell plot for chronoamperogram findings, (**B**) slope of the plot of straight lines versus hydrazine content.

Chronoamperometry was employed to evaluate the catalytic hydrazine oxidation on the modified electrode surface in the presence of different hydrazine concentrations on the working electrode set at the potential value of 850 mV (Figure 4). The hydrazine diffusion coefficient was also determined. According to previous findings, the electrochemical current of hydrazine under the mass transport-limited condition could be calculated using the Cottrell method (Equation (2)):

I = nFA D^1/2^ C_b_ π^−1/2^ t^−1/2^ (2)

In this equation, n is the number of electrons, F is the Faraday constant, A is the area of the electrode, while D and C_b_ stand for diffusion coefficient (cm^2^/s) and bulk concentration (mol/cm^3^), respectively. Inset A of Figure 4 shows the plot of I versus t^−1/2^ based on experiments for various hydrazine specimens. Inset B of Figure 4 displays the slope of the straight lines versus hydrazine content. The D value for hydrazine was calculated to be 1.31 × 10^−5^ cm^2^/s based on Cottrell equation and the obtained slopes. This value is comparable with the values reported in previous works (2.5 × 10^−5^ cm^2^/s [47] and 8.3 × 10^−5^ cm^2^/s [48]).

### 3.5. Calibration Curve and Limit of Detection

**Figure 5:**
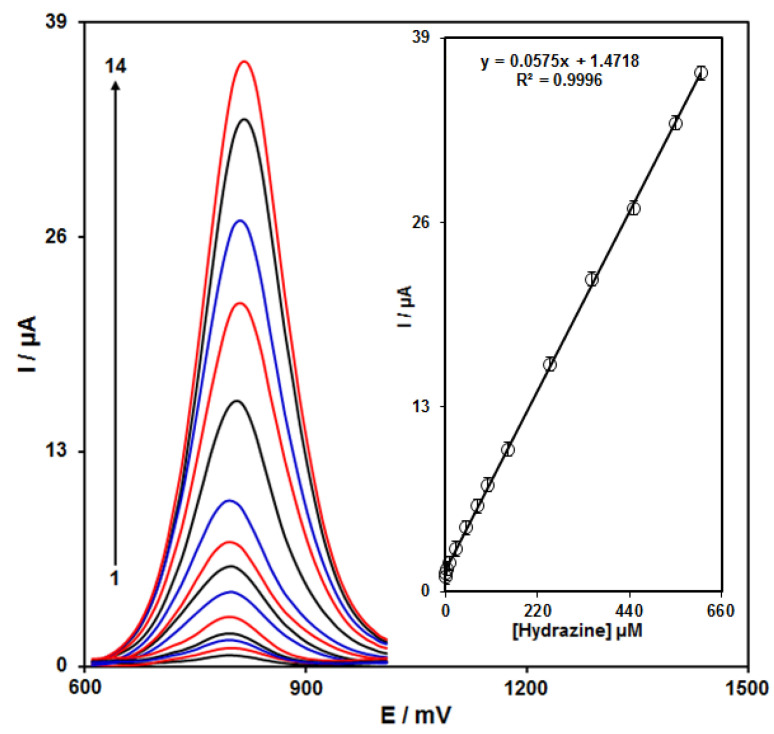
DPVs of ZnFe_2_O_4_/RGO/SPE in the presence of 0.1 M PBS at the pH value of 7.0 for detection of hydrazine at different concentrations, indicated by numbers 1–14 corresponding to 0.03, 0.3, 3.0, 10.0, 25.0, 50.0, 75.0, 100.0, 150.0, 250.0, 350.0, 450.0, 550.0, and 610.0 µM of hydrazine. Inset: plot of peak current as a function of different hydrazine concentrations (0.03–610.0 µM).

The peak currents of hydrazine electro-oxidation on the ZnFe_2_O_4_/RGO/SPE surface were used for the hydrazine detection. Hypersensitivity and appropriate analytical features are the advantages of differential pulse voltammetry (DPV); hence, different hydrazine concentrations in the ZnFe_2_O_4_/RGO/SPE and PBS (0.1 M) were used for DPV analysis as shown in Figure 5. The peak currents of hydrazine oxidation on the surface of ZnFe_2_O_4_/RGO/SPE depends linearly on hydrazine concentrations (0.03 to 610.0 μM). The linear equation was as y = 0.0575X + 1.4718, the correlation coefficient was estimated 0.9996, and the limit of detection (with three signal-to-noise ratio) was estimated at 0.01 µM using following equation:

Limit of detection = 3 s_b_/m (3)

In the above equation, m is the slope of the calibration plot (0.0575 μA μM^−1^), and s_b_ is the standard deviation of the blank response obtained from 20 replicate measurements of the blank solution. The LOD and linear range of hydrazine at ZnFe_2_O_4_/RGO/SPE presented in this work were compared with the reported modified electrodes and are shown in Table 1. As shown in Table 1, the prepared ZnFe_2_O_4_/RGO/SPE exhibited a lower LOD together with a wide linear dynamic range compared with previously reported works for the voltammetric determination of hydrazine.

### 3.6. Interference Studies

Interference studies were investigated to know how the results for the hydrazine analysis are affected by the presence of various inorganic ions and organic compounds. According to the used definition, the tolerance limit was defined as the ratio of the concentration of the interfering species to the hydrazine (50.0 μM), which led to a relative error of less than ±5.0%. The possible interference was investigated by the addition of various ions and biological compounds such as Mg^2+^, Na^+^, Ca^2+^, Cl^−^, NO_3_^−^ (200 fold excess), glucose, sucrose, ascorbic acid, uric acid, urea, L-cystine, and dopamine (50 fold excess) to PBS (pH 7.0) in the presence of 50.0 μM hydrazine. It was found that the addition of these interfering species has no remarkable effect on the DPV signal of hydrazine. These results indicate that the modified electrode has good selectivity for hydrazine determination.

### 3.7. Real Sample Analysis

The fabricated ZnFe_2_O_4_/RGO/SPE was used for the detection of hydrazine present in varied water specimens using the method of standard additions. The hydrazine concentration and recovery rate are shown in Table 2. An excellent recovery rate was found for the hydrazine, and the mean relative standard deviation (R.S.D. %) confirmed the reproducibility. The applicability of ZnFe2O4/RGO/SPE sensor was confirmed by sensitively detection of hydrazine concentrations in drinking, tap, and river water specimens in the presence of 0.1 M PBS.

## 4. Conclusions

We developed a new ZnFe_2_O_4_/RGO nanocomposite-modified SPE for hydrazine detection, the results of which showed a significant improvement of electrochemical sensitivity of hydrazine on the proposed electrode when compared to the bare SPE, due to rapid electron transfer and enhanced conductivity. The as-produced modified electrode was successfully applied for the hydrazine detection in real specimens because of the excellent selectivity and sensitivity of voltammetric responses, low limit of detection (0.01 µM), simple preparation, and surface regeneration.

## Figures and Tables

**Table 1:** Comparison of the efficiency of the ZnFe_2_O_4_/RGO/SPE electrode with the literature modified electrodes for hydrazine determination.

Electrochemical Sensor	Electrochemical Method	Linear Range	LOD	Ref.
Nitrogen-doped graphene -polyvinylpyrrolidone-gold nanoparticles/screen-printed carbon electrode	Square wave voltammetry	2–300 μM	0.07 μM	[3]
1-benzyl-4-ferrocenyl-1H- [1,2,3]-triazole/carbon nanotube modified glassy carbon electrode	Square wave voltammetry	0.5–700.0 µM	33.0 nM	[47]
poly(vinyl alcohol)/chitosan/TiO2/chlorophyll nanocomposite modified screen printed electrode	DPV	0.45–350.0 μM	0.015 μM	[48]
polythiophene-ZnO nanocomposite/glassy carbon electrode	Amperometry	0.5–48 μM	0.207 μM	[49]
Copper sulfide–ordered mesoporous carbon/glassy carbon electrode	Amperometry	0.25–40 μM	0.10 μM	[50]
Chrysanthemum-like Co_3_O_4_/glassy carbon electrode	Amperometry	50–1088 μM	3.7 μM	[51]
ZnFe_2_O_4_/RGO/SPE	DPV	0.03–610.0 µM	0.01 μM	This work

**Table 2:** Recoveries for detection of hydrazine in water specimens in the presence of 0.1 M PBS. (*n* = 3).

Sample	Spiked	Found	Recovery (%)	R.S.D. (%)
Drinking water	0	-	-	-
	4.0	4.1	102.5	1.7
	6.0	5.8	96.7	3.3
	8.0	8.3	103.7	2.3
	10.0	9.9	99.0	2.9
Tap water	0	-	-	-
	5.0	4.9	98.0	2.2
	8.0	8.1	101.2	1.8
	11.0	11.4	103.6	3.5
	14.0	13.9	99.2	2.1
River water	0	-	-	-
	4.0	3.0	97.5	3.6
	7.0	7.1	101.4	2.1
	10.0	9.8	98.0	2.9
	13.0	13.5	103.8	2.5

## Data Availability

The data presented in this study are available on request from the corresponding authors.

## Supplementary Materials

The following supporting information can be downloaded at: https://www.mdpi.com/article/10.3390/nano12030491/s1, Figure S1: XRD patterns (a), SEM images (b), and TEM images for ZnFe_2_O_4_-RGO (c).
