# Supplementary material for: Electrochemical Sensor Based on ZnFe2O4/RGO Nanocomposite for Ultrasensitive Detection of Hydrazine in Real Samples
Source: Nanomaterials (Basel). 2022 Jan 29;12(3):491. doi: 10.3390/nano12030491 (PMC8838434; doi:10.3390/nano12030491)
Supplement: Supplementary file 1 [file nanomaterials-12-00491-s001.zip › nanomaterials-1548714-supplementary.pdf]

Supplementary Materials

Electrochemical Sensor Based on $ZnFe_2O_4$/RGO Nanocomposite for Ultrasensitive Detection of Hydrazine in Real Samples

Somayeh Tajik [1], Mohammad Bagher Askari [2], Sayed Ali Ahmadi [3], Fraiba Garkani Nejad [4], Zahra Dourandish [4], Razieh Razavi [5], Hadi Beitollahi [2,*] and Antonio Di Bartolomeo [6,*]

[1] Research Center of Tropical and Infectious Diseases, Kerman University of Medical Sciences, Kerman P.O. Box 76169-13555, Iran; s.tajik@kmu.ac.ir
[2] Environment Department, Institute of Science and High Technology and Environmental Sciences, Graduate University of Advanced Technology, Kerman P.O. Box 76318-85356, Iran; mbaskari@phd.guilan.ac.ir
[3] Department of Chemistry, Kerman Branch, Islamic Azad University, Kerman P.O. Box 763151-31167, Iran; saahmadi@iauk.ac.ir
[4] Department of Chemistry, Faculty of Science, Shahid Bahonar University of Kerman, Kerman P.O. Box 76175-133, Iran; f.garkani95@gmail.com (F.G.N.); z.dourandish2017@gmail.com (Z.D.)
[5] Department of Chemistry, Faculty of Science, University of Jiroft, Jiroft P.O. Box 78671-55311, Iran; R.Razavi@ujiroft.ac.ir
[6] Department of Physics "E.R. Caianaiello", University of Salerno, 84084 Fisciano, Italy
* Correspondence: h.beitollahi@kgut.ac.ir (H.B.); adibartolomeo@unisa.it (A.D.B.)

Supplementary Materials

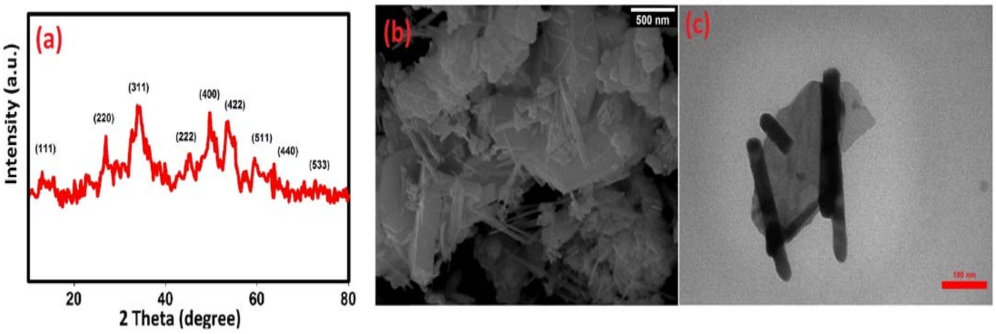

Figure S1. XRD patterns (a), SEM images (b), and TEM images for $ZnFe_2O_4$-RGO (c).

Stability, repeatability, and reproducibility

The long-term stability experiment using DPV was also performed by keeping the $ZnFe_2O_4$/RGO/SPE in the ambient conditions. The results showed that the peak current of 60.0 μM hydrazine the modified electrode stayed at 99.1% of its primary current after 5 days, 98.1% after 10 days, and 96.5% after 15 days, indicating the excellent long-term stability of the suggested sensor. To validate the repeatability of the $ZnFe_2O_4$/RGO/SPE, electro-oxidation of 60.0 μM hydrazine on same electrode was studied by 10 replicate voltammetric measurements and the repeatability was found to be excellent with relative standard deviation (RSD) 3.3%.

Furthermore, five $ZnFe_2O_4$/RGO/SPE were prepared under the same conditions to compare the response currents of 60.0 μM carmoisine. The value of RSD of the response currents was 3.8%, which demonstrated that the modified electrode has excellent reproducibility.
